# Bis[μ-*N*′-(2-methyl-1-oxidopropanyl­idene)-2-oxidobenzohydrazidato]tetra­pyridine­trinickel(II)

**DOI:** 10.1107/S1600536811034362

**Published:** 2011-08-27

**Authors:** Xiao-Hua Chen, Chun-Ling Xie, Ming-Xing Yang, Li-Juan Chen

**Affiliations:** a College of Chemistry and Materials Science, Fujian Normal University, Fuzhou, Fujian 350007, People’s Republic of China

## Abstract

The asymmetric unit of the title trinuclear Ni^II^ compound, [Ni_3_(C_11_H_11_N_2_O_3_)(C_5_H_5_N)_4_], contains two independent mol­ecules which are located on individual inversion centres. The central Ni atom, located on an inversion centre, is coordinated by two pyridine N atoms and is further *N*,*O*-chelated by two *N*-(2-methyl­propano­yl)salicyloylhydrazidate anions in an elongated octa­hedral coordination geometry. The terminal Ni atom is coordinated by a pyridine ligand and is further *N*,*N*′,*O*-chelated by an *N*-(2-methyl­propano­yl)salicyloyl­hydrazidate anion in a distorted square-planar coordination geometry. Weak intra­molecular C—H⋯O hydrogen bonding is observed in the structure.

## Related literature

For general background to *N*-acyl-salicylhydrazide ligands and their metal complexes, see: Chen *et al.* (2011 ▶); Dou *et al.* (2006 ▶); John *et al.* (2005 ▶); Li *et al.* (2005 ▶); Lin *et al.* (2007 ▶); Luo *et al.* (2007 ▶); Luo *et al.* (2008 ▶); Xiao *et al.* (2007 ▶); Yang *et al.* (2005 ▶). For related structures, see: Xiao & Jin (2008 ▶); Yang *et al.* (2003 ▶). For the synthesis, see: Yang *et al.* (2003 ▶).
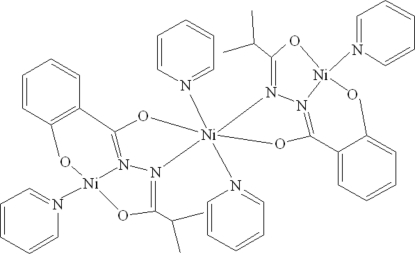


## Experimental

### 

#### Crystal data

[Ni_3_(C_11_H_11_N_2_O_3_)(C_5_H_5_N)_4_]
*M* *_r_* = 930.97
Triclinic, 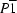

*a* = 9.866 (3) Å
*b* = 12.325 (5) Å
*c* = 18.240 (7) Å
α = 109.324 (15)°
β = 96.474 (13)°
γ = 93.516 (13)°
*V* = 2068.2 (13) Å^3^
*Z* = 2
Mo *K*α radiation
μ = 1.41 mm^−1^
*T* = 293 K
0.46 × 0.26 × 0.14 mm

#### Data collection

Rigaku R-AXIS RAPID diffractometer
Absorption correction: multi-scan *TEXRAY* (Molecular Structure Corporation, 1999 ▶) *T*_min_ = 0.651, *T*_max_ = 0.821
20490 measured reflections
9360 independent reflections
5875 reflections with *I* > 2σ(*I*)
*R*_int_ = 0.051

#### Refinement

*R*[*F* ^2^ > 2σ(*F* ^2^)] = 0.050
*wR*(*F* ^2^) = 0.104
*S* = 1.04
9360 reflections
539 parameters
H-atom parameters constrained
Δρ_max_ = 0.37 e Å^−3^
Δρ_min_ = −0.46 e Å^−3^

### 

Data collection: *TEXRAY* (Molecular Structure Corporation, 1999 ▶); cell refinement: *TEXRAY*; data reduction: *TEXSAN* (Molecular Structure Corporation, 1999 ▶); program(s) used to solve structure: *SHELXS97* (Sheldrick, 2008 ▶); program(s) used to refine structure: *SHELXL97* (Sheldrick, 2008 ▶); molecular graphics: *ORTEX* (McArdle, 1995 ▶); software used to prepare material for publication: *SHELXL97*.

## Supplementary Material

Crystal structure: contains datablock(s) I, global. DOI: 10.1107/S1600536811034362/xu5275sup1.cif

Structure factors: contains datablock(s) I. DOI: 10.1107/S1600536811034362/xu5275Isup2.hkl

Additional supplementary materials:  crystallographic information; 3D view; checkCIF report

## Figures and Tables

**Table 1:** Selected bond lengths (Å)

Ni1—O1	1.808 (2)
Ni1—N1	1.828 (2)
Ni1—O3	1.841 (2)
Ni1—N3	1.943 (3)
Ni2—O2	2.032 (2)
Ni2—N2	2.076 (2)
Ni2—N4	2.146 (3)
Ni3—O4	1.815 (3)
Ni3—N5	1.823 (3)
Ni3—O6	1.845 (2)
Ni3—N7	1.934 (3)
Ni4—O5	2.018 (2)
Ni4—N6	2.060 (3)
Ni4—N8	2.169 (3)

**Table 2:** Hydrogen-bond geometry (Å, °)

*D*—H⋯*A*	*D*—H	H⋯*A*	*D*⋯*A*	*D*—H⋯*A*
C9—H9*A*⋯O2^i^	0.98	2.43	3.332 (5)	152
C30—H30*A*⋯O5^ii^	0.98	2.38	3.272 (5)	152

Symmetry codes: (i) 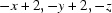; (ii) 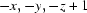.

**Fig. 1.**
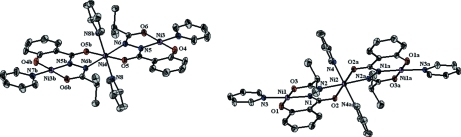
A view of the asymmetric unit of the title complex, showing 20% probability displacement ellipsoids for non-H atoms.

## supplementary crystallographic information

### Comment

In the recent years, much attention has been paid to the coordination chemistry of the trianionic pentadentate N-acyl-salicylhydrazide ligands and their metal complexes. These kinds of pentadentate ligands have been utilized in the system of self-assembly in metallacrowns with different ring-sizes and nuclearities based on trivalent 3d metal ions such as Fe(III), Gd(III), Co(III) and Mn(III) (Dou *et al.*, 2006; John *et al.*, 2005; Li *et al.*, 2005; Xiao *et al.*, 2007), and a few trinuclear complexes based on bivalent 3d metal ions such as Ni(II), Cu(II) and Zn(II) (Chen *et al.*, 2011; Lin *et al.*, 2007; Luo *et al.*, 2007; Luo *et al.*, 2008; Yang *et al.*, 2005). Some of these complexes have potential application in chemically modified electrodes, anion-selective separation agents, magnetic materials and biological activities.

There are two crystallographically independent molecules of (I) in the asymmetric unit (Fig. 1). Each independent molecule is composed of three Ni(II) ions, two L^3-^ and four pyridine molecules. The ligand serves as both bidentate for the central Ni(II) ion and, at the same time, tridentate for the two terminal Ni(II) ions, forming a linear trinuclear nickel structure. The neighboring Ni···Ni interatomic distances are 4.605 (2)Å and 4.589 (2)Å, respectively. The coordination geometry of the three Ni(II) atoms in each trinuclear molecule follows a square-planar/octahedral/square-planar mode. The central Ni(II) atom located on the crystallographic inversion is six-coordinated by two pyridine N atoms in axial positions, and the two hydrazine N atoms and carbonyl O atoms of two ligands in the equatorial plane, conferring an elongated octahedral geometry. Each basal plane of the two octahedra is ideally planar and each Ni(II) ion complexly lies in the equatorial plane. The terminal Ni(II) atom is coordinated in a square-planar configuration composed of the other hydrazine nitrogen carbonyl oxygen and phenolic oxygen of one ligand, as well as one pyridine N atom. The distances in the coordination planes around the Ni(II) ions (Table 1) and the bond lengths in the ligand moieties are comparable with the related Ni(II) complexes based on the similar pentadentate N-acyl-salicylhydrazide ligands (Xiao & Jin 2008; Yang *et al.*, 2003).

### Experimental

The ligand N-(2-methylpropanoyl)salicylhydrazide (H_3_L) was prepared according to the reported procedure reported by Yang *et al.* (2003). Five drops of pyridine were added dropwise to the mixture of [Ni(OAc)_2_].4H_2_O (24.8 mg, 0.1 mmol) in methanol (5 ml) and H_3_L (24.8 mg, 0.1 mmol) in DMF (5 ml). The resulting red solution was further stirred for 1 h and filtered. The red crystals separated after several days were collected. Analysis calculated for C_42_H_42_N_8_O_6_Ni_3_ (%): C, 54.19; H, 4.55; N, 12.04. Found: C, 55.36; H, 4.91; N, 11.82.

### Refinement

All H atoms were placed at calculated positions and treated as riding on their parent atoms with C—H = 0.93-0.98 Å , and U_iso_(H) = 1.5U_eq_(C) for methyl H atoms and 1.2U_eq_(C) for the others.

### Crystal data

**Table:**

[Ni_3_(C_11_H_11_N_2_O_3_)(C_5_H_5_N)_4_]	*Z* = 2
*M**_r_* = 930.97	*F*(000) = 964
Triclinic, *P*1	*D*_x_ = 1.495 Mg m^−^^3^
Hall symbol: -P 1	Mo *K*α radiation, λ = 0.71073 Å
*a* = 9.866 (3) Å	Cell parameters from 5875 reflections
*b* = 12.325 (5) Å	θ = 3.1–27.5°
*c* = 18.240 (7) Å	µ = 1.41 mm^−^^1^
α = 109.324 (15)°	*T* = 293 K
β = 96.474 (13)°	Prism, red
γ = 93.516 (13)°	0.46 × 0.26 × 0.14 mm
*V* = 2068.2 (13) Å^3^	

### Data collection

**Table:**

Rigaku R-AXIS RAPID diffractometer	9360 independent reflections
Radiation source: fine-focus sealed tube	5875 reflections with *I* > 2σ(*I*)
graphite	*R*_int_ = 0.051
ω scans	θ_max_ = 27.5°, θ_min_ = 3.1°
Absorption correction: multi-scan *TEXRAY* (Molecular Structure Corporation, 1999)	*h* = −12→11
*T*_min_ = 0.651, *T*_max_ = 0.821	*k* = −15→15
20490 measured reflections	*l* = −23→23

### Refinement

**Table:**

Refinement on *F*^2^	Primary atom site location: structure-invariant direct methods
Least-squares matrix: full	Secondary atom site location: difference Fourier map
*R*[*F*^2^ > 2σ(*F*^2^)] = 0.050	Hydrogen site location: inferred from neighbouring sites
*wR*(*F*^2^) = 0.104	H-atom parameters constrained
*S* = 1.04	*w* = 1/[σ^2^(*F*_o_^2^) + (0.0318*P*)^2^ + 0.8559*P*] where *P* = (*F*_o_^2^ + 2*F*_c_^2^)/3
9360 reflections	(Δ/σ)_max_ = 0.001
539 parameters	Δρ_max_ = 0.37 e Å^−^^3^
0 restraints	Δρ_min_ = −0.46 e Å^−^^3^

### Special details

**Table:**

Geometry. All esds (except the esd in the dihedral angle between two l.s. planes) are estimated using the full covariance matrix. The cell esds are taken into account individually in the estimation of esds in distances, angles and torsion angles; correlations between esds in cell parameters are only used when they are defined by crystal symmetry. An approximate (isotropic) treatment of cell esds is used for estimating esds involving l.s. planes.
Refinement. Refinement of F^2^ against ALL reflections. The weighted R-factor wR and goodness of fit S are based on F^2^, conventional R-factors R are based on F, with F set to zero for negative F^2^. The threshold expression of F^2^ > 2sigma(F^2^) is used only for calculating R-factors(gt) etc. and is not relevant to the choice of reflections for refinement. R-factors based on F^2^ are statistically about twice as large as those based on F, and R- factors based on ALL data will be even larger.

### Fractional atomic coordinates and isotropic or equivalent isotropic displacement parameters (Å^2^)

**Table:**

	*x*	*y*	*z*	*U*_iso_*/*U*_eq_	
Ni1	0.93957 (4)	0.66089 (3)	0.05241 (2)	0.03894 (12)	
Ni2	1.0000	1.0000	0.0000	0.03734 (15)	
Ni3	−0.06516 (5)	0.32923 (4)	0.44143 (3)	0.05115 (14)	
Ni4	0.0000	0.0000	0.5000	0.04078 (16)	
O1	0.7678 (2)	0.5919 (2)	0.01168 (15)	0.0569 (7)	
O2	0.8335 (2)	0.88568 (18)	−0.05727 (13)	0.0420 (5)	
O3	1.1140 (2)	0.73331 (19)	0.09258 (14)	0.0468 (6)	
O4	0.0721 (3)	0.3594 (2)	0.39034 (17)	0.0677 (8)	
O5	0.1519 (2)	0.0692 (2)	0.45856 (14)	0.0476 (6)	
O6	−0.1979 (2)	0.3001 (2)	0.49806 (15)	0.0572 (7)	
N1	0.9243 (3)	0.7769 (2)	0.01130 (15)	0.0374 (6)	
N2	1.0431 (2)	0.8583 (2)	0.03276 (15)	0.0378 (6)	
N3	0.9702 (3)	0.5467 (2)	0.10306 (16)	0.0427 (7)	
N4	0.8942 (3)	1.0555 (2)	0.10051 (16)	0.0419 (7)	
N5	0.0080 (3)	0.2072 (2)	0.46030 (16)	0.0447 (7)	
N6	−0.0704 (3)	0.1552 (2)	0.50322 (16)	0.0435 (7)	
N7	−0.1622 (4)	0.4484 (3)	0.41838 (19)	0.0596 (8)	
N8	−0.1065 (3)	−0.0751 (2)	0.38106 (17)	0.0484 (7)	
C1	0.6842 (3)	0.6149 (3)	−0.0418 (2)	0.0451 (8)	
C2	0.7023 (3)	0.7135 (3)	−0.06517 (19)	0.0395 (8)	
C3	0.6044 (3)	0.7264 (3)	−0.1220 (2)	0.0526 (9)	
H3A	0.6150	0.7918	−0.1365	0.063*	
C4	0.4933 (4)	0.6470 (4)	−0.1574 (2)	0.0647 (11)	
H4A	0.4300	0.6579	−0.1955	0.078*	
C5	0.4767 (4)	0.5501 (4)	−0.1356 (2)	0.0650 (11)	
H5A	0.4021	0.4948	−0.1597	0.078*	
C6	0.5686 (4)	0.5351 (3)	−0.0793 (2)	0.0590 (11)	
H6A	0.5546	0.4699	−0.0651	0.071*	
C7	0.8246 (3)	0.7977 (3)	−0.03513 (19)	0.0376 (7)	
C8	1.1369 (3)	0.8261 (3)	0.07424 (19)	0.0389 (8)	
C9	1.2773 (3)	0.8907 (3)	0.1018 (2)	0.0502 (9)	
H9A	1.2785	0.9612	0.0882	0.060*	
C10	1.3833 (4)	0.8170 (4)	0.0608 (3)	0.0841 (14)	
H10A	1.3609	0.7974	0.0050	0.126*	
H10B	1.3833	0.7475	0.0737	0.126*	
H10C	1.4726	0.8596	0.0778	0.126*	
C11	1.3106 (5)	0.9247 (4)	0.1904 (3)	0.0884 (15)	
H11A	1.2415	0.9695	0.2149	0.133*	
H11B	1.3984	0.9698	0.2078	0.133*	
H11C	1.3130	0.8563	0.2044	0.133*	
C12	0.8723 (4)	0.4652 (3)	0.0996 (2)	0.0598 (11)	
H12A	0.7873	0.4614	0.0706	0.072*	
C13	0.8922 (5)	0.3859 (4)	0.1375 (3)	0.0742 (13)	
H13A	0.8215	0.3300	0.1337	0.089*	
C14	1.0156 (5)	0.3904 (3)	0.1804 (2)	0.0666 (12)	
H14A	1.0297	0.3396	0.2078	0.080*	
C15	1.1185 (4)	0.4712 (3)	0.1824 (2)	0.0595 (10)	
H15A	1.2053	0.4744	0.2095	0.071*	
C16	1.0912 (4)	0.5474 (3)	0.1436 (2)	0.0556 (10)	
H16A	1.1614	0.6028	0.1458	0.067*	
C17	0.7578 (4)	1.0526 (3)	0.0959 (2)	0.0589 (10)	
H17A	0.7054	1.0243	0.0465	0.071*	
C18	0.6920 (4)	1.0892 (4)	0.1602 (3)	0.0709 (12)	
H18A	0.5969	1.0864	0.1544	0.085*	
C19	0.7665 (5)	1.1297 (4)	0.2327 (3)	0.0807 (14)	
H19A	0.7234	1.1555	0.2772	0.097*	
C20	0.9062 (5)	1.1318 (4)	0.2392 (2)	0.0721 (12)	
H20A	0.9599	1.1582	0.2882	0.087*	
C21	0.9648 (4)	1.0943 (3)	0.1721 (2)	0.0550 (10)	
H21A	1.0599	1.0961	0.1769	0.066*	
C22	0.1843 (4)	0.3066 (4)	0.3802 (2)	0.0629 (11)	
C23	0.2116 (3)	0.2087 (3)	0.4006 (2)	0.0486 (9)	
C24	0.3333 (4)	0.1602 (4)	0.3849 (2)	0.0651 (11)	
H24A	0.3507	0.0952	0.3982	0.078*	
C25	0.4290 (5)	0.2053 (5)	0.3502 (3)	0.0870 (15)	
H25A	0.5096	0.1714	0.3400	0.104*	
C26	0.4023 (5)	0.3012 (5)	0.3312 (3)	0.0936 (18)	
H26A	0.4667	0.3332	0.3086	0.112*	
C27	0.2836 (5)	0.3508 (4)	0.3446 (3)	0.0805 (15)	
H27A	0.2678	0.4149	0.3300	0.097*	
C28	0.1207 (3)	0.1579 (3)	0.4412 (2)	0.0446 (8)	
C29	−0.1747 (4)	0.2131 (3)	0.5209 (2)	0.0469 (8)	
C30	−0.2695 (4)	0.1822 (3)	0.5716 (2)	0.0574 (10)	
H30A	−0.2430	0.1121	0.5810	0.069*	
C31	−0.2554 (5)	0.2809 (4)	0.6499 (3)	0.0929 (16)	
H31A	−0.1615	0.2951	0.6742	0.139*	
H31B	−0.3124	0.2602	0.6836	0.139*	
H31C	−0.2834	0.3494	0.6413	0.139*	
C32	−0.4159 (4)	0.1598 (4)	0.5316 (3)	0.0933 (17)	
H32A	−0.4219	0.1016	0.4807	0.140*	
H32B	−0.4452	0.2299	0.5261	0.140*	
H32C	−0.4738	0.1335	0.5626	0.140*	
C33	−0.1188 (6)	0.5050 (4)	0.3733 (3)	0.1002 (18)	
H33A	−0.0362	0.4888	0.3541	0.120*	
C34	−0.1897 (7)	0.5859 (5)	0.3537 (4)	0.119 (2)	
H34A	−0.1563	0.6220	0.3210	0.143*	
C35	−0.3087 (7)	0.6130 (4)	0.3823 (3)	0.1012 (18)	
H35A	−0.3583	0.6678	0.3700	0.121*	
C36	−0.3529 (6)	0.5582 (4)	0.4291 (3)	0.0996 (17)	
H36A	−0.4343	0.5745	0.4497	0.120*	
C37	−0.2764 (5)	0.4775 (4)	0.4463 (3)	0.0839 (14)	
H37A	−0.3077	0.4416	0.4796	0.101*	
C38	−0.2429 (4)	−0.0946 (4)	0.3670 (3)	0.0684 (12)	
H38A	−0.2918	−0.0726	0.4094	0.082*	
C39	−0.3142 (5)	−0.1448 (5)	0.2939 (3)	0.0911 (16)	
H39A	−0.4095	−0.1555	0.2867	0.109*	
C40	−0.2437 (6)	−0.1797 (4)	0.2305 (3)	0.0912 (16)	
H40A	−0.2897	−0.2164	0.1800	0.109*	
C41	−0.1053 (6)	−0.1588 (4)	0.2441 (3)	0.0875 (15)	
H41A	−0.0543	−0.1800	0.2026	0.105*	
C42	−0.0414 (4)	−0.1066 (4)	0.3187 (2)	0.0658 (11)	
H42A	0.0537	−0.0920	0.3267	0.079*	

### Atomic displacement parameters (Å^2^)

**Table:**

	*U*^11^	*U*^22^	*U*^33^	*U*^12^	*U*^13^	*U*^23^
Ni1	0.0456 (2)	0.0334 (2)	0.0421 (3)	0.00117 (18)	0.0072 (2)	0.0187 (2)
Ni2	0.0422 (3)	0.0316 (3)	0.0408 (4)	0.0003 (3)	0.0040 (3)	0.0169 (3)
Ni3	0.0656 (3)	0.0429 (3)	0.0497 (3)	0.0040 (2)	0.0041 (2)	0.0237 (2)
Ni4	0.0432 (3)	0.0403 (4)	0.0451 (4)	0.0076 (3)	0.0130 (3)	0.0201 (3)
O1	0.0585 (15)	0.0538 (16)	0.0673 (18)	−0.0101 (12)	0.0003 (13)	0.0379 (14)
O2	0.0445 (13)	0.0391 (13)	0.0465 (14)	−0.0006 (10)	−0.0009 (11)	0.0234 (11)
O3	0.0523 (14)	0.0388 (13)	0.0552 (15)	0.0005 (11)	0.0004 (12)	0.0267 (12)
O4	0.0761 (19)	0.0680 (19)	0.076 (2)	0.0011 (15)	0.0115 (16)	0.0484 (17)
O5	0.0478 (13)	0.0465 (14)	0.0569 (16)	0.0083 (11)	0.0153 (12)	0.0253 (13)
O6	0.0648 (16)	0.0490 (15)	0.0714 (18)	0.0188 (12)	0.0162 (14)	0.0340 (14)
N1	0.0375 (14)	0.0339 (15)	0.0429 (16)	−0.0018 (12)	0.0035 (12)	0.0175 (13)
N2	0.0389 (15)	0.0324 (14)	0.0448 (17)	−0.0011 (12)	0.0051 (13)	0.0177 (13)
N3	0.0523 (17)	0.0351 (15)	0.0453 (17)	0.0042 (13)	0.0110 (14)	0.0188 (14)
N4	0.0492 (17)	0.0356 (15)	0.0436 (17)	0.0054 (13)	0.0110 (14)	0.0157 (14)
N5	0.0521 (17)	0.0436 (17)	0.0454 (17)	0.0042 (14)	0.0116 (14)	0.0229 (15)
N6	0.0471 (16)	0.0395 (16)	0.0506 (18)	0.0070 (13)	0.0136 (14)	0.0216 (15)
N7	0.080 (2)	0.0431 (18)	0.056 (2)	0.0084 (17)	−0.0012 (18)	0.0200 (17)
N8	0.0557 (18)	0.0441 (17)	0.0493 (19)	0.0114 (14)	0.0107 (15)	0.0188 (15)
C1	0.0464 (19)	0.044 (2)	0.048 (2)	−0.0010 (16)	0.0098 (17)	0.0199 (18)
C2	0.0375 (17)	0.0420 (19)	0.0411 (19)	0.0028 (15)	0.0068 (15)	0.0166 (16)
C3	0.045 (2)	0.061 (2)	0.058 (2)	−0.0060 (18)	0.0025 (18)	0.030 (2)
C4	0.050 (2)	0.082 (3)	0.063 (3)	−0.012 (2)	−0.008 (2)	0.035 (2)
C5	0.050 (2)	0.074 (3)	0.068 (3)	−0.019 (2)	−0.002 (2)	0.028 (2)
C6	0.056 (2)	0.060 (2)	0.064 (3)	−0.0168 (19)	0.004 (2)	0.031 (2)
C7	0.0415 (18)	0.0346 (18)	0.0384 (19)	0.0018 (14)	0.0082 (15)	0.0142 (16)
C8	0.0448 (19)	0.0353 (18)	0.0393 (19)	0.0053 (15)	0.0044 (15)	0.0165 (16)
C9	0.0428 (19)	0.046 (2)	0.067 (3)	−0.0044 (16)	−0.0047 (18)	0.030 (2)
C10	0.051 (2)	0.081 (3)	0.124 (4)	0.008 (2)	0.014 (3)	0.039 (3)
C11	0.081 (3)	0.102 (4)	0.074 (3)	−0.023 (3)	−0.023 (3)	0.037 (3)
C12	0.060 (2)	0.052 (2)	0.078 (3)	0.0016 (19)	0.011 (2)	0.035 (2)
C13	0.076 (3)	0.061 (3)	0.105 (4)	−0.003 (2)	0.018 (3)	0.054 (3)
C14	0.092 (3)	0.054 (3)	0.070 (3)	0.015 (2)	0.015 (2)	0.040 (2)
C15	0.075 (3)	0.049 (2)	0.059 (3)	0.006 (2)	0.001 (2)	0.027 (2)
C16	0.068 (2)	0.041 (2)	0.062 (3)	0.0006 (18)	0.000 (2)	0.027 (2)
C17	0.057 (2)	0.061 (3)	0.061 (3)	0.006 (2)	0.008 (2)	0.024 (2)
C18	0.060 (3)	0.074 (3)	0.085 (4)	0.014 (2)	0.028 (3)	0.028 (3)
C19	0.093 (4)	0.079 (3)	0.072 (3)	0.005 (3)	0.043 (3)	0.020 (3)
C20	0.087 (3)	0.077 (3)	0.045 (2)	−0.007 (3)	0.009 (2)	0.013 (2)
C21	0.062 (2)	0.056 (2)	0.050 (2)	0.0016 (19)	0.012 (2)	0.021 (2)
C22	0.074 (3)	0.071 (3)	0.048 (2)	−0.016 (2)	0.002 (2)	0.032 (2)
C23	0.048 (2)	0.057 (2)	0.041 (2)	−0.0063 (18)	0.0063 (17)	0.0203 (19)
C24	0.058 (2)	0.081 (3)	0.062 (3)	−0.001 (2)	0.019 (2)	0.030 (2)
C25	0.070 (3)	0.113 (4)	0.089 (4)	−0.003 (3)	0.035 (3)	0.043 (3)
C26	0.081 (3)	0.136 (5)	0.078 (3)	−0.024 (3)	0.017 (3)	0.059 (4)
C27	0.085 (3)	0.100 (4)	0.071 (3)	−0.020 (3)	0.005 (3)	0.056 (3)
C28	0.048 (2)	0.045 (2)	0.040 (2)	−0.0051 (17)	0.0057 (16)	0.0152 (17)
C29	0.056 (2)	0.040 (2)	0.049 (2)	0.0121 (17)	0.0105 (18)	0.0181 (18)
C30	0.059 (2)	0.047 (2)	0.076 (3)	0.0194 (19)	0.029 (2)	0.027 (2)
C31	0.132 (4)	0.076 (3)	0.076 (3)	0.022 (3)	0.049 (3)	0.021 (3)
C32	0.056 (3)	0.097 (4)	0.144 (5)	0.017 (3)	0.035 (3)	0.055 (4)
C33	0.135 (5)	0.093 (4)	0.112 (4)	0.033 (3)	0.035 (4)	0.076 (4)
C34	0.167 (6)	0.108 (5)	0.130 (5)	0.054 (4)	0.040 (5)	0.089 (4)
C35	0.141 (5)	0.065 (3)	0.106 (5)	0.029 (3)	−0.009 (4)	0.046 (3)
C36	0.110 (4)	0.076 (3)	0.131 (5)	0.036 (3)	0.014 (4)	0.055 (4)
C37	0.103 (4)	0.064 (3)	0.100 (4)	0.021 (3)	0.013 (3)	0.047 (3)
C38	0.061 (3)	0.084 (3)	0.064 (3)	0.005 (2)	0.005 (2)	0.031 (3)
C39	0.074 (3)	0.118 (4)	0.084 (4)	−0.008 (3)	−0.014 (3)	0.049 (4)
C40	0.121 (4)	0.088 (4)	0.054 (3)	−0.015 (3)	−0.017 (3)	0.023 (3)
C41	0.108 (4)	0.089 (4)	0.055 (3)	0.010 (3)	0.013 (3)	0.010 (3)
C42	0.067 (3)	0.075 (3)	0.052 (3)	0.013 (2)	0.010 (2)	0.014 (2)

### Geometric parameters (Å, °)

**Table:**

Ni1—O1	1.808 (2)	C11—H11B	0.9600
Ni1—N1	1.828 (2)	C11—H11C	0.9600
Ni1—O3	1.841 (2)	C12—C13	1.382 (5)
Ni1—N3	1.943 (3)	C12—H12A	0.9300
Ni2—O2	2.032 (2)	C13—C14	1.358 (6)
Ni2—O2^i^	2.032 (2)	C13—H13A	0.9300
Ni2—N2^i^	2.076 (2)	C14—C15	1.366 (5)
Ni2—N2	2.076 (2)	C14—H14A	0.9300
Ni2—N4	2.146 (3)	C15—C16	1.373 (5)
Ni2—N4^i^	2.146 (3)	C15—H15A	0.9300
Ni3—O4	1.815 (3)	C16—H16A	0.9300
Ni3—N5	1.823 (3)	C17—C18	1.363 (5)
Ni3—O6	1.845 (2)	C17—H17A	0.9300
Ni3—N7	1.934 (3)	C18—C19	1.356 (6)
Ni4—O5	2.018 (2)	C18—H18A	0.9300
Ni4—O5^ii^	2.018 (2)	C19—C20	1.367 (6)
Ni4—N6	2.060 (3)	C19—H19A	0.9300
Ni4—N6^ii^	2.060 (3)	C20—C21	1.366 (5)
Ni4—N8	2.169 (3)	C20—H20A	0.9300
Ni4—N8^ii^	2.169 (3)	C21—H21A	0.9300
O1—C1	1.321 (4)	C22—C23	1.409 (5)
O2—C7	1.278 (3)	C22—C27	1.410 (5)
O3—C8	1.308 (3)	C23—C24	1.391 (5)
O4—C22	1.318 (5)	C23—C28	1.461 (4)
O5—C28	1.279 (4)	C24—C25	1.380 (5)
O6—C29	1.296 (4)	C24—H24A	0.9300
N1—C7	1.318 (4)	C25—C26	1.369 (7)
N1—N2	1.423 (3)	C25—H25A	0.9300
N2—C8	1.293 (4)	C26—C27	1.364 (7)
N3—C16	1.329 (4)	C26—H26A	0.9300
N3—C12	1.331 (4)	C27—H27A	0.9300
N4—C21	1.327 (4)	C29—C30	1.503 (5)
N4—C17	1.336 (4)	C30—C32	1.509 (5)
N5—C28	1.325 (4)	C30—C31	1.525 (6)
N5—N6	1.425 (3)	C30—H30A	0.9800
N6—C29	1.298 (4)	C31—H31A	0.9600
N7—C37	1.310 (5)	C31—H31B	0.9600
N7—C33	1.329 (5)	C31—H31C	0.9600
N8—C42	1.326 (5)	C32—H32A	0.9600
N8—C38	1.333 (5)	C32—H32B	0.9600
C1—C6	1.407 (5)	C32—H32C	0.9600
C1—C2	1.421 (4)	C33—C34	1.370 (6)
C2—C3	1.392 (4)	C33—H33A	0.9300
C2—C7	1.467 (4)	C34—C35	1.354 (7)
C3—C4	1.366 (5)	C34—H34A	0.9300
C3—H3A	0.9300	C35—C36	1.342 (6)
C4—C5	1.386 (5)	C35—H35A	0.9300
C4—H4A	0.9300	C36—C37	1.379 (6)
C5—C6	1.359 (5)	C36—H36A	0.9300
C5—H5A	0.9300	C37—H37A	0.9300
C6—H6A	0.9300	C38—C39	1.359 (6)
C8—C9	1.499 (4)	C38—H38A	0.9300
C9—C11	1.520 (5)	C39—C40	1.376 (7)
C9—C10	1.524 (5)	C39—H39A	0.9300
C9—H9A	0.9800	C40—C41	1.353 (6)
C10—H10A	0.9600	C40—H40A	0.9300
C10—H10B	0.9600	C41—C42	1.357 (6)
C10—H10C	0.9600	C41—H41A	0.9300
C11—H11A	0.9600	C42—H42A	0.9300
			
O1—Ni1—N1	94.86 (11)	C9—C11—H11A	109.5
O1—Ni1—O3	178.73 (10)	C9—C11—H11B	109.5
N1—Ni1—O3	83.88 (10)	H11A—C11—H11B	109.5
O1—Ni1—N3	90.25 (11)	C9—C11—H11C	109.5
N1—Ni1—N3	174.59 (12)	H11A—C11—H11C	109.5
O3—Ni1—N3	91.01 (10)	H11B—C11—H11C	109.5
O2—Ni2—O2^i^	180.000 (1)	N3—C12—C13	122.6 (4)
O2—Ni2—N2^i^	101.40 (9)	N3—C12—H12A	118.7
O2^i^—Ni2—N2^i^	78.60 (9)	C13—C12—H12A	118.7
O2—Ni2—N2	78.60 (9)	C14—C13—C12	119.5 (4)
O2^i^—Ni2—N2	101.40 (9)	C14—C13—H13A	120.2
N2^i^—Ni2—N2	180.00 (13)	C12—C13—H13A	120.2
O2—Ni2—N4	88.41 (10)	C13—C14—C15	118.7 (3)
O2^i^—Ni2—N4	91.59 (10)	C13—C14—H14A	120.7
N2^i^—Ni2—N4	93.00 (10)	C15—C14—H14A	120.7
N2—Ni2—N4	87.00 (10)	C14—C15—C16	118.6 (4)
O2—Ni2—N4^i^	91.59 (10)	C14—C15—H15A	120.7
O2^i^—Ni2—N4^i^	88.41 (10)	C16—C15—H15A	120.7
N2^i^—Ni2—N4^i^	87.00 (10)	N3—C16—C15	123.8 (3)
N2—Ni2—N4^i^	93.00 (10)	N3—C16—H16A	118.1
N4—Ni2—N4^i^	180.000 (1)	C15—C16—H16A	118.1
O4—Ni3—N5	94.82 (12)	N4—C17—C18	123.0 (4)
O4—Ni3—O6	176.94 (12)	N4—C17—H17A	118.5
N5—Ni3—O6	83.73 (11)	C18—C17—H17A	118.5
O4—Ni3—N7	89.84 (14)	C19—C18—C17	119.4 (4)
N5—Ni3—N7	173.64 (13)	C19—C18—H18A	120.3
O6—Ni3—N7	91.82 (13)	C17—C18—H18A	120.3
O5—Ni4—O5^ii^	180.0	C18—C19—C20	118.8 (4)
O5—Ni4—N6	78.90 (10)	C18—C19—H19A	120.6
O5^ii^—Ni4—N6	101.10 (10)	C20—C19—H19A	120.6
O5—Ni4—N6^ii^	101.10 (10)	C21—C20—C19	118.4 (4)
O5^ii^—Ni4—N6^ii^	78.90 (10)	C21—C20—H20A	120.8
N6—Ni4—N6^ii^	180.0	C19—C20—H20A	120.8
O5—Ni4—N8	89.05 (10)	N4—C21—C20	123.9 (4)
O5^ii^—Ni4—N8	90.95 (10)	N4—C21—H21A	118.1
N6—Ni4—N8	88.16 (11)	C20—C21—H21A	118.1
N6^ii^—Ni4—N8	91.84 (11)	O4—C22—C23	125.2 (3)
O5—Ni4—N8^ii^	90.95 (10)	O4—C22—C27	117.2 (4)
O5^ii^—Ni4—N8^ii^	89.05 (10)	C23—C22—C27	117.6 (4)
N6—Ni4—N8^ii^	91.84 (11)	C24—C23—C22	119.1 (3)
N6^ii^—Ni4—N8^ii^	88.16 (11)	C24—C23—C28	117.7 (3)
N8—Ni4—N8^ii^	180.0	C22—C23—C28	123.1 (4)
C1—O1—Ni1	126.08 (19)	C25—C24—C23	122.1 (4)
C7—O2—Ni2	112.94 (19)	C25—C24—H24A	119.0
C8—O3—Ni1	112.0 (2)	C23—C24—H24A	119.0
C22—O4—Ni3	126.6 (2)	C26—C25—C24	118.4 (5)
C28—O5—Ni4	113.2 (2)	C26—C25—H25A	120.8
C29—O6—Ni3	111.7 (2)	C24—C25—H25A	120.8
C7—N1—N2	114.7 (2)	C27—C26—C25	121.5 (4)
C7—N1—Ni1	131.7 (2)	C27—C26—H26A	119.2
N2—N1—Ni1	113.58 (18)	C25—C26—H26A	119.2
C8—N2—N1	109.5 (2)	C26—C27—C22	121.2 (4)
C8—N2—Ni2	140.3 (2)	C26—C27—H27A	119.4
N1—N2—Ni2	109.56 (18)	C22—C27—H27A	119.4
C16—N3—C12	116.8 (3)	O5—C28—N5	121.8 (3)
C16—N3—Ni1	120.9 (2)	O5—C28—C23	120.0 (3)
C12—N3—Ni1	122.3 (2)	N5—C28—C23	118.2 (3)
C21—N4—C17	116.5 (3)	O6—C29—N6	121.9 (3)
C21—N4—Ni2	119.9 (2)	O6—C29—C30	118.3 (3)
C17—N4—Ni2	123.6 (3)	N6—C29—C30	119.7 (3)
C28—N5—N6	114.2 (3)	C29—C30—C32	110.6 (3)
C28—N5—Ni3	131.7 (2)	C29—C30—C31	109.0 (3)
N6—N5—Ni3	114.1 (2)	C32—C30—C31	110.4 (4)
C29—N6—N5	108.5 (3)	C29—C30—H30A	108.9
C29—N6—Ni4	140.6 (2)	C32—C30—H30A	108.9
N5—N6—Ni4	109.83 (19)	C31—C30—H30A	108.9
C37—N7—C33	115.9 (4)	C30—C31—H31A	109.5
C37—N7—Ni3	121.4 (3)	C30—C31—H31B	109.5
C33—N7—Ni3	122.7 (3)	H31A—C31—H31B	109.5
C42—N8—C38	116.2 (4)	C30—C31—H31C	109.5
C42—N8—Ni4	122.8 (3)	H31A—C31—H31C	109.5
C38—N8—Ni4	121.0 (3)	H31B—C31—H31C	109.5
O1—C1—C6	117.4 (3)	C30—C32—H32A	109.5
O1—C1—C2	125.3 (3)	C30—C32—H32B	109.5
C6—C1—C2	117.3 (3)	H32A—C32—H32B	109.5
C3—C2—C1	118.6 (3)	C30—C32—H32C	109.5
C3—C2—C7	119.0 (3)	H32A—C32—H32C	109.5
C1—C2—C7	122.3 (3)	H32B—C32—H32C	109.5
C4—C3—C2	122.6 (3)	N7—C33—C34	123.3 (5)
C4—C3—H3A	118.7	N7—C33—H33A	118.3
C2—C3—H3A	118.7	C34—C33—H33A	118.3
C3—C4—C5	118.8 (4)	C35—C34—C33	119.5 (5)
C3—C4—H4A	120.6	C35—C34—H34A	120.3
C5—C4—H4A	120.6	C33—C34—H34A	120.3
C6—C5—C4	120.4 (3)	C36—C35—C34	118.0 (5)
C6—C5—H5A	119.8	C36—C35—H35A	121.0
C4—C5—H5A	119.8	C34—C35—H35A	121.0
C5—C6—C1	122.2 (3)	C35—C36—C37	119.4 (5)
C5—C6—H6A	118.9	C35—C36—H36A	120.3
C1—C6—H6A	118.9	C37—C36—H36A	120.3
O2—C7—N1	122.2 (3)	N7—C37—C36	123.8 (5)
O2—C7—C2	119.3 (3)	N7—C37—H37A	118.1
N1—C7—C2	118.5 (3)	C36—C37—H37A	118.1
N2—C8—O3	121.0 (3)	N8—C38—C39	123.3 (4)
N2—C8—C9	122.1 (3)	N8—C38—H38A	118.4
O3—C8—C9	116.8 (3)	C39—C38—H38A	118.4
C8—C9—C11	110.1 (3)	C38—C39—C40	119.3 (5)
C8—C9—C10	110.0 (3)	C38—C39—H39A	120.4
C11—C9—C10	111.0 (3)	C40—C39—H39A	120.4
C8—C9—H9A	108.6	C41—C40—C39	117.9 (5)
C11—C9—H9A	108.6	C41—C40—H40A	121.1
C10—C9—H9A	108.6	C39—C40—H40A	121.1
C9—C10—H10A	109.5	C40—C41—C42	119.4 (5)
C9—C10—H10B	109.5	C40—C41—H41A	120.3
H10A—C10—H10B	109.5	C42—C41—H41A	120.3
C9—C10—H10C	109.5	N8—C42—C41	123.9 (4)
H10A—C10—H10C	109.5	N8—C42—H42A	118.0
H10B—C10—H10C	109.5	C41—C42—H42A	118.0
			
N1—Ni1—O1—C1	11.6 (3)	O1—C1—C2—C3	−179.4 (3)
O3—Ni1—O1—C1	3(6)	C6—C1—C2—C3	1.2 (5)
N3—Ni1—O1—C1	−170.2 (3)	O1—C1—C2—C7	4.5 (5)
O2^i^—Ni2—O2—C7	36 (100)	C6—C1—C2—C7	−174.8 (3)
N2^i^—Ni2—O2—C7	−168.4 (2)	C1—C2—C3—C4	−1.5 (6)
N2—Ni2—O2—C7	11.6 (2)	C7—C2—C3—C4	174.7 (4)
N4—Ni2—O2—C7	−75.6 (2)	C2—C3—C4—C5	0.5 (6)
N4^i^—Ni2—O2—C7	104.4 (2)	C3—C4—C5—C6	0.7 (6)
O1—Ni1—O3—C8	10 (6)	C4—C5—C6—C1	−0.9 (7)
N1—Ni1—O3—C8	1.2 (2)	O1—C1—C6—C5	−179.5 (4)
N3—Ni1—O3—C8	−177.0 (2)	C2—C1—C6—C5	−0.1 (6)
N5—Ni3—O4—C22	−3.7 (3)	Ni2—O2—C7—N1	−9.4 (4)
O6—Ni3—O4—C22	58 (2)	Ni2—O2—C7—C2	173.7 (2)
N7—Ni3—O4—C22	−179.4 (3)	N2—N1—C7—O2	−1.6 (4)
O5^ii^—Ni4—O5—C28	171 (100)	Ni1—N1—C7—O2	179.5 (2)
N6—Ni4—O5—C28	−12.1 (2)	N2—N1—C7—C2	175.4 (3)
N6^ii^—Ni4—O5—C28	167.9 (2)	Ni1—N1—C7—C2	−3.6 (5)
N8—Ni4—O5—C28	76.2 (2)	C3—C2—C7—O2	5.3 (5)
N8^ii^—Ni4—O5—C28	−103.8 (2)	C1—C2—C7—O2	−178.7 (3)
O4—Ni3—O6—C29	−62 (2)	C3—C2—C7—N1	−171.8 (3)
N5—Ni3—O6—C29	−0.4 (2)	C1—C2—C7—N1	4.3 (5)
N7—Ni3—O6—C29	175.1 (3)	N1—N2—C8—O3	−2.0 (4)
O1—Ni1—N1—C7	−3.2 (3)	Ni2—N2—C8—O3	167.0 (2)
O3—Ni1—N1—C7	176.7 (3)	N1—N2—C8—C9	176.4 (3)
N3—Ni1—N1—C7	−163.9 (11)	Ni2—N2—C8—C9	−14.6 (6)
O1—Ni1—N1—N2	177.9 (2)	Ni1—O3—C8—N2	0.2 (4)
O3—Ni1—N1—N2	−2.3 (2)	Ni1—O3—C8—C9	−178.3 (2)
N3—Ni1—N1—N2	17.1 (14)	N2—C8—C9—C11	125.0 (4)
C7—N1—N2—C8	−176.2 (3)	O3—C8—C9—C11	−56.6 (4)
Ni1—N1—N2—C8	2.9 (3)	N2—C8—C9—C10	−112.4 (4)
C7—N1—N2—Ni2	11.2 (3)	O3—C8—C9—C10	66.0 (4)
Ni1—N1—N2—Ni2	−169.66 (12)	C16—N3—C12—C13	1.6 (6)
O2—Ni2—N2—C8	179.1 (4)	Ni1—N3—C12—C13	−178.0 (3)
O2^i^—Ni2—N2—C8	−0.9 (4)	N3—C12—C13—C14	0.2 (7)
N2^i^—Ni2—N2—C8	−56 (100)	C12—C13—C14—C15	−2.3 (7)
N4—Ni2—N2—C8	−91.9 (4)	C13—C14—C15—C16	2.6 (6)
N4^i^—Ni2—N2—C8	88.1 (4)	C12—N3—C16—C15	−1.2 (6)
O2—Ni2—N2—N1	−11.92 (18)	Ni1—N3—C16—C15	178.3 (3)
O2^i^—Ni2—N2—N1	168.08 (18)	C14—C15—C16—N3	−0.8 (6)
N2^i^—Ni2—N2—N1	112 (100)	C21—N4—C17—C18	1.3 (5)
N4—Ni2—N2—N1	77.06 (19)	Ni2—N4—C17—C18	−179.4 (3)
N4^i^—Ni2—N2—N1	−102.94 (19)	N4—C17—C18—C19	−0.7 (6)
O1—Ni1—N3—C16	177.1 (3)	C17—C18—C19—C20	−0.5 (7)
N1—Ni1—N3—C16	−22.1 (14)	C18—C19—C20—C21	0.8 (7)
O3—Ni1—N3—C16	−2.8 (3)	C17—N4—C21—C20	−1.0 (5)
O1—Ni1—N3—C12	−3.5 (3)	Ni2—N4—C21—C20	179.7 (3)
N1—Ni1—N3—C12	157.4 (12)	C19—C20—C21—N4	−0.1 (6)
O3—Ni1—N3—C12	176.7 (3)	Ni3—O4—C22—C23	6.7 (6)
O2—Ni2—N4—C21	149.5 (3)	Ni3—O4—C22—C27	−174.5 (3)
O2^i^—Ni2—N4—C21	−30.5 (3)	O4—C22—C23—C24	178.6 (4)
N2^i^—Ni2—N4—C21	−109.2 (3)	C27—C22—C23—C24	−0.2 (6)
N2—Ni2—N4—C21	70.8 (3)	O4—C22—C23—C28	−4.4 (6)
N4^i^—Ni2—N4—C21	−95 (100)	C27—C22—C23—C28	176.9 (4)
O2—Ni2—N4—C17	−29.8 (3)	C22—C23—C24—C25	0.4 (6)
O2^i^—Ni2—N4—C17	150.2 (3)	C28—C23—C24—C25	−176.8 (4)
N2^i^—Ni2—N4—C17	71.6 (3)	C23—C24—C25—C26	0.2 (7)
N2—Ni2—N4—C17	−108.4 (3)	C24—C25—C26—C27	−1.1 (8)
N4^i^—Ni2—N4—C17	86 (100)	C25—C26—C27—C22	1.3 (8)
O4—Ni3—N5—C28	−1.3 (3)	O4—C22—C27—C26	−179.5 (4)
O6—Ni3—N5—C28	−178.6 (3)	C23—C22—C27—C26	−0.7 (7)
N7—Ni3—N5—C28	135.8 (11)	Ni4—O5—C28—N5	9.5 (4)
O4—Ni3—N5—N6	178.9 (2)	Ni4—O5—C28—C23	−171.7 (2)
O6—Ni3—N5—N6	1.6 (2)	N6—N5—C28—O5	1.9 (5)
N7—Ni3—N5—N6	−44.1 (13)	Ni3—N5—C28—O5	−177.9 (2)
C28—N5—N6—C29	177.7 (3)	N6—N5—C28—C23	−177.0 (3)
Ni3—N5—N6—C29	−2.4 (3)	Ni3—N5—C28—C23	3.2 (5)
C28—N5—N6—Ni4	−11.8 (3)	C24—C23—C28—O5	−2.5 (5)
Ni3—N5—N6—Ni4	168.04 (13)	C22—C23—C28—O5	−179.6 (3)
O5—Ni4—N6—C29	178.2 (4)	C24—C23—C28—N5	176.5 (3)
O5^ii^—Ni4—N6—C29	−1.8 (4)	C22—C23—C28—N5	−0.6 (5)
N6^ii^—Ni4—N6—C29	15.9 (4)	Ni3—O6—C29—N6	−1.1 (4)
N8—Ni4—N6—C29	88.8 (4)	Ni3—O6—C29—C30	176.7 (3)
N8^ii^—Ni4—N6—C29	−91.2 (4)	N5—N6—C29—O6	2.3 (5)
O5—Ni4—N6—N5	12.52 (19)	Ni4—N6—C29—O6	−163.5 (3)
O5^ii^—Ni4—N6—N5	−167.48 (19)	N5—N6—C29—C30	−175.5 (3)
N6^ii^—Ni4—N6—N5	−149.8 (4)	Ni4—N6—C29—C30	18.7 (6)
N8—Ni4—N6—N5	−76.9 (2)	O6—C29—C30—C32	56.5 (5)
N8^ii^—Ni4—N6—N5	103.1 (2)	N6—C29—C30—C32	−125.7 (4)
O4—Ni3—N7—C37	−175.2 (4)	O6—C29—C30—C31	−65.0 (5)
N5—Ni3—N7—C37	47.6 (14)	N6—C29—C30—C31	112.8 (4)
O6—Ni3—N7—C37	2.2 (4)	C37—N7—C33—C34	−2.5 (8)
O4—Ni3—N7—C33	5.3 (4)	Ni3—N7—C33—C34	177.0 (5)
N5—Ni3—N7—C33	−131.9 (12)	N7—C33—C34—C35	1.5 (10)
O6—Ni3—N7—C33	−177.2 (4)	C33—C34—C35—C36	−0.2 (10)
O5—Ni4—N8—C42	30.4 (3)	C34—C35—C36—C37	0.1 (9)
O5^ii^—Ni4—N8—C42	−149.6 (3)	C33—N7—C37—C36	2.4 (7)
N6—Ni4—N8—C42	109.3 (3)	Ni3—N7—C37—C36	−177.1 (4)
N6^ii^—Ni4—N8—C42	−70.7 (3)	C35—C36—C37—N7	−1.3 (9)
N8^ii^—Ni4—N8—C42	129 (100)	C42—N8—C38—C39	0.8 (6)
O5—Ni4—N8—C38	−151.1 (3)	Ni4—N8—C38—C39	−177.8 (4)
O5^ii^—Ni4—N8—C38	28.9 (3)	N8—C38—C39—C40	1.1 (7)
N6—Ni4—N8—C38	−72.2 (3)	C38—C39—C40—C41	−2.0 (8)
N6^ii^—Ni4—N8—C38	107.8 (3)	C39—C40—C41—C42	1.0 (8)
N8^ii^—Ni4—N8—C38	−52 (100)	C38—N8—C42—C41	−1.8 (6)
Ni1—O1—C1—C6	165.5 (3)	Ni4—N8—C42—C41	176.7 (3)
Ni1—O1—C1—C2	−13.8 (5)	C40—C41—C42—N8	0.9 (8)

Symmetry codes: (i) −*x*+2, −*y*+2, −*z*; (ii) −*x*, −*y*, −*z*+1.

### Hydrogen-bond geometry (Å, °)

**Table:**

*D*—H···*A*	*D*—H	H···*A*	*D*···*A*	*D*—H···*A*
C9—H9A···O2^i^	0.98	2.43	3.332 (5)	152
C30—H30A···O5^ii^	0.98	2.38	3.272 (5)	152

Symmetry codes: (i) −*x*+2, −*y*+2, −*z*; (ii) −*x*, −*y*, −*z*+1.
